# Investigating the association between human brainstem microstructural integrity and hypertension using magnetic resonance relaxometry

**DOI:** 10.1038/s41440-025-02114-1

**Published:** 2025-01-23

**Authors:** John P. Laporte, Mohammad A. B. S. Akhonda, Luis E. Cortina, Mary E. Faulkner, Zhaoyuan Gong, Alex Guo, Jonghyun Bae, Noam Y. Fox, Nathan Zhang, Christopher M. Bergeron, Luigi Ferrucci, Josephine M. Egan, Mustapha Bouhrara

**Affiliations:** 1 Laboratory of Clinical Investigation, National Institute on Aging, National Institutes of Health, Baltimore, 21224 MD USA; 2 Translational Gerontology Branch, National Institute on Aging, National Institutes of Health, Baltimore, 21224 MD USA

**Keywords:** Brainstem, Hypertension, Myelin, Magnetic resonance imaging

## Abstract

The brainstem plays a vital role in regulating blood pressure, and disruptions to its neural pathways have been linked to hypertension. However, it remains unclear whether subtle microstructural changes in the brainstem are associated with an individual’s blood pressure status. This exploratory, cross-sectional study investigated the relationship between brainstem microstructure, myelination, and hypertensive status in 116 cognitively unimpaired adults (aged 22–94 years). Advanced MRI techniques, including relaxometry (R1, R2) and myelin water fraction (MWF) analysis, were employed to assess microstructural integrity and myelin content in ten brainstem subregions. Our results revealed significant associations between higher microstructural damage or lower myelin content (indicated by lower R1, R2, or MWF values) and hypertensive status, particularly in the midbrain tegmentum. Notably, combining these MRI metrics yielded high classification accuracy (AUC > 0.85). Our findings suggest a potential link between disrupted brainstem tissue integrity, myelin content, and elevated blood pressure, warranting further longitudinal investigations to explore this relationship.

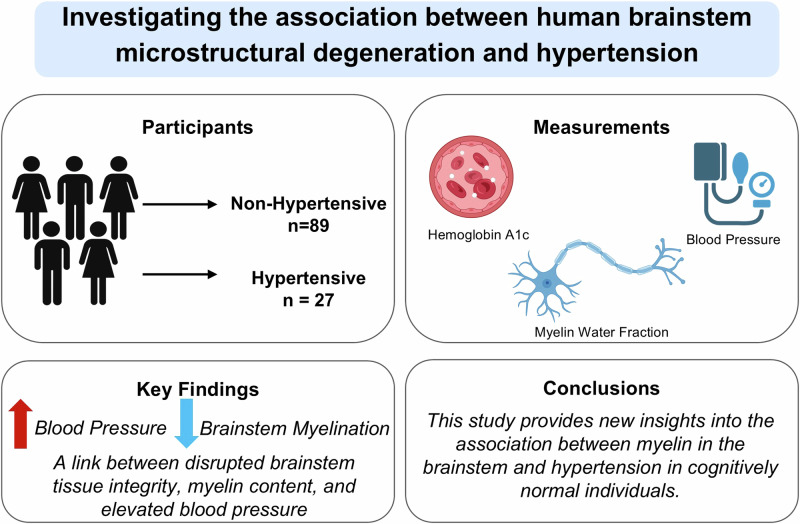

## Introduction

Hypertension, or elevated blood pressure, is a leading risk factor for severe cardiovascular and neurological disorders, including coronary artery disease, strokes, vascular dementia, and Alzheimer’s disease [1–5]. The brainstem, a primary regulator of blood pressure within the autonomic nervous system, plays a crucial role in integrating signals from various brain regions, the autonomic nervous system, and the periphery to maintain blood pressure homeostasis [6, 7]. Disruptions to this complex network may impair blood pressure regulation, contributing to hypertension [8]. Exploratory case studies have reported complete resolution of brainstem lesions following antihypertensive treatment [9]. Additionally, other studies have identified associations between white matter hyperintensities and hypertension, as revealed through agnetic resonance imaging (MRI) [10]. While severe brainstem lesions are related to acute hypertension, it remains unclear whether subtle, subclinical damage may also contribute to elevated blood pressure. Therefore, investigating the relationship between brainstem microstructural integrity and blood pressure may uncover underlying mechanisms and potential connections, advancing our understanding of hypertension and revealing novel therapeutic targets.

Previous studies using MRI, primarily diffusion tensor imaging (DTI), have shown an association between hypertension and abnormal cerebral microstructural integrity [11–13]. Recent investigations have also used multicomponent relaxometry to explore the relationship between myelin content and hypertension in aging adults [14]. However, these studies have mainly focused on the cerebrum. Investigating the brainstem is challenging due to its small size, requiring high-spatial resolution MR imaging to minimize partial volume effects [15]. Despite this, studies using DTI and brainstem volume measurements have reported negative associations between brainstem volume/neuronal fiber integrity with blood pressure [16, 17].

Quantitative MRI (qMRI) techniques have been developed for high-spatial resolution mapping of longitudinal and transverse relaxation rates (R_1_, R_2_) in complex CNS structures including the spinal cord, choroid plexus, and brainstem [15–28]. R_1_ and R_2_ provide valuable information on cerebral microstructural integrity, composition, and biochemistry [29], and are sensitive to capturing tissue deterioration, even at early stages. Multicomponent relaxometry can also quantify myelin content through myelin water fraction (MWF) measurement, which is a more specific surrogate of myelin content than relaxation rates [30–32]. MWF has been used in studies of brain maturation and degeneration [32–38]. The Bayesian Monte Carlo Analysis of multicomponent driven equilibrium steady-state observation of T_1_ and T_2_ (BMC-mcDESPOT) has enabled high-spatial resolution mapping of MWF [39–42] and has been used to investigate risk factors affecting myelination and age/sex-associated differences in brainstem myelination [14, 38, 43–50]. Recently, it has shown a direct correlation between brainstem myelination and motor function [18, 21].

Using BMC-mcDESPOT for MWF, R_1_, and R_2_ mapping, we examined the association between brainstem microstructural integrity, including myelin content, and hypertensive status in 116 cognitively unimpaired adults (aged 22–94 years). We also investigated the accuracy of these MRI metrics for classifying hypertensive vs. control participants. This study aimed to explore the potential implication of cerebral microstructural and myelin degeneration in the brainstem on blood pressure dysregulation.

## Material & methods

### Study cohort

Participants are volunteers of the Baltimore Longitudinal Study of Aging (BLSA) and the Genetic and Epigenetic Signatures of Translational Aging Laboratory Testing (GESTALT) studies [51, 52]. Both BLSA and GESTALT evaluate multiple biomarkers associated with aging, with virtually identical inclusion and exclusion criteria. Participants with metallic implants or major neurologic or medical disorders are excluded on first admission. All participants were administered the Mini Mental State Examination (MMSE). Informed consent was obtained from participants prior to participation in compliance with NIH Institutional Review Board.

### Data acquisition

MRI scans were performed on a 3 T whole body Philips MRI system (Achieva, Best, The Netherlands) using the internal quadrature body coil for transmission and an eight-channel phased-array head coil for reception. Each participant underwent our BMC-mcDESPOT protocol for MWF, *R*_*1*_, and *R*_*2*_ mapping [42, 44]. This imaging protocol consisted of 3D spoiled gradient recalled echo (SPGR) images acquired with flip angles (FAs) of [2 4 6 8 10 12 14 16 18 20]°, echo time (TE) of 1.37 ms, repetition time (TR) of 5 ms and acquisition time of ~5 min, as well as 3D balanced steady-state free precession (bSSFP) images acquired with FAs of [2 4 7 11 16 24 32 40 50 60]°, TE of 2.8 ms, TR of 5.8 ms, and acquisition time of ~6 min. The bSSFP images were acquired with radiofrequency (RF) excitation pulse phase increments of 0 or 180° to account for off-resonance effects, with a total scan time of ~12 min (~6 min for each phase-cycling scan). All SPGR and bSSFP images were acquired with an acquisition matrix of 150 × 130 × 94, voxel size 1.6 mm × 1.6 mm × 1.6 mm. To correct for excitation RF inhomogeneity [53], we used the double-angle method (DAM), which consisted of acquiring two fast spin-echo images with FAs of 45° and 90°, TE of 102 ms, TR of 3000 ms, acquisition voxel size of 2.6 mm × 2.6 mm × 4 mm, and acquisition time of ~4 min. The total acquisition time was ~21 min. All images were acquired with field-of-view of 240 mm × 208 mm × 150 mm, SENSE factor of 2, and reconstructed to a voxel size of 1 mm × 1 mm × 1 mm. We emphasize that all MRI studies and ancillary measurements were performed with the same MRI system, with the same pulse sequences, and at the same facility for both BLSA and GESTALT participants.

### Data processing

For each participant, using the FLIRT analysis as implemented in the FSL software [50], all SPGR, bSSFP and DAM images were linearly registered to the SPGR image obtained at FA of 8° and the respective derived transformation matrices were then applied to the original SPGR, bSSFP, and DAM images. Then, a whole-brain MWF map was generated using BMC-mcDESPOT from these co-registered SPGR, bSSFP, and DAM datasets [40–42]. BMC-mcDESPOT assumes a two-relaxation time components system consisting of a short component, attributed to myelin water, and a long component, attributed to intra- and extracellular water. We used the signal model explicitly accounting for non-zero TE [40–42]. This emerging method offers rapid and reliable high-spatial resolution whole-brain MWF map within feasible clinical time [39–42, 46, 47], and has been used in assessing myelin loss in mild cognitive impairment and dementias, as well as examining factors influencing cerebral myelination in normative aging [44, 45, 49, 54–56]. A whole-brain *R*_*1*_ map was also generated from the co-registered SPGR and DAM datasets using DESPOT1 [57], and a whole-brain *R*_*2*_ map was generated from the co-registered bSSFP and DAM datasets using DESPOT2 [57].

Further, using FSL software [58], the averaged SPGR image over FAs underwent nonlinear registration to the Montreal Neurological Institute (MNI) standard space, and the computed transformation matrix was then applied to the MWF, *R*_*1*_, and *R*_*2*_ maps. Ten regions of interest (ROIs) were defined from the MNI structural atlas corresponding to the superior cerebellar peduncle (SCP), middle cerebellar peduncle (MCP), inferior cerebellar peduncle (ICP), cerebral peduncle (CP), medulla (MED), midbrain (MID), pons (PON), red nucleus (RN), substantia nigra (SN), and subthalamic nucleus (STH). ROIs were eroded to reduce partial volume effect. Within each ROI, the mean MWF, *R*_*1*_, and *R*_*2*_ values were calculated.

### Systolic and diastolic blood pressure

Systolic and diastolic blood pressures (SBP and DBP) were recorded three times in both arms in a seated position using a mercury sphygmomanometer sized to the arm of each participant, and the mean of the systolic and diastolic measurements were used in the subsequent analyses [51]. Hypertensive status was defined as a systolic blood pressure greater than or equal to 140 mmHg, a diastolic blood pressure greater than or equal to 90 mmHg, or the use of anti-hypertension medications by participants.

### HbA1c

HbA1c, or hemoglobin A1c, is a measure of average blood glucose levels over the past 3 months and is commonly used to assess long-term glycemic control, particularly in individuals with diabetes. HbA1c was measured on a whole blood sample using an Abbott Afinion 2© analyzer (Series #AF220006881).

### Statistical analyses

To investigate the potential implication of brainstem microstructural deterioration, including myelin degeneration, on hypertensive status, we conducted logistic regression analyses using the hypertensive status as the dependent variable and age, HbA1c, and MWF, *R*_*1*_ or *R*_*2*_ values within each ROI as the independent variables. In all cases, the threshold for statistical significance was *p* < 0.05, while close-to-significance was taken as *p* < 0.1.

Further, for each MRI metric and brainstem subregion/ROI, we calculated the receiver operating characteristic curves (ROC-curves). These curves provide a graphical illustration of how our MRI metrics can accurately classify hypertensive cases, and to further examine the association between brainstem health and hypertension. We also calculated the area under the curve (AUC) which offers a single scalar value to evaluate the model’s discriminative power in distinguishing between hypertensive and non-hypertensive groups based on our MRI metrics alone. Moreover, we evaluated whether combining these MRI metrics improves classification accuracy with and without incorporating demographic information.

All calculations were performed with MATLAB software (MathWorks, Natick, MA, USA).

## Results

### Participants demographic characteristics

The demographic characteristics of the study participants are summarized in Table 1. The participants’ ages ranged from 22 to 94 years, with a mean age of 56.1 ± 20.7 years. The cohort consisted of 65 males (56.0%) and 51 females (44.0%), with 35 participants (30.2%) identifying as cigarette smokers and 77 (66.4%) as nonsmokers. Additionally, 27 individuals (23.3%) had hypertension, and 23 (85.2%) of these participants were taking antihypertensive medication. After excluding 9 participants due to cognitive impairment, 4 due to missing blood pressure data, and 6 due to low image quality primarily caused by severe motion artifacts, our final study cohort consisted of 116 cognitively unimpaired participants. The demographics table is split into Hypertensive, Control, and Total groups to facilitate comparisons between groups. The participants’ Mini-Mental State Examination (MMSE) score was 28.81 ± 1.393, indicating normal cognitive function. Their mean systolic blood pressure (SBP) was 116.12 ± 13.99 mmHg, and their mean HbA1c level was 5.53 ± 0.50. The lipid profiles showed mean values of 97.63 ± 53.47 for triglycerides, 178.16 ± 38.03 for cholesterol, 63.57 ± 23.93 for high-density lipoprotein (HDL), and 97.64 ± 33.44 for low-density lipoprotein (LDL). Only age, HbA1c, and SBP differed significantly between the Control and Hypertensive groups. Consequently, age and HbA1c were used as covariates in the logistic regression analysis. SBP was not included due to its confounding role in defining hypertension while also exhibiting non-significant association with MRI metrics.

**Table 1:** Demographic characteristics of participants of the study cohort

	Total Sample *N* = 116		
	Hypertensive	Non-Hypertensive	Total
Hypertensive status, *N*(%)	27 *(23.3%)*	89 *(76.7%)*	116 *(100%)*
Age (yrs.), mean ± SD (*min-max***)**	74.04 ± 15.61 *(39–94)*	50.64 ± 18.94 *(22–94)*	56.09 ± 20.69 *(22–94)*
MMSE, mean ± SD (*min-max***)**	28.30 ± 1.66 *(23–30)*	28.97 ± 1.26 *(25–30)*	28.81 ± 1.39 *(23–30)*
SBP (mmHg), mean ± SD (*min-max***)**	125.78 ± 15.69 *(106–161)*	113.19 ± 12.07 *(89–139)*	116.12 ± 13.99 *(89–161)*
Sex			
Male, *N*(%)	16 *(59.3%)*	49 *(55.1%)*	65 *(56.0%)*
Female, *N*(%)	11 *(40.7%)*	40 *(44.9%)*	51*(44.0%)*
Smoking status			
Smokers, *N*(%)	15 *(55.6%)*	62 *(69.7%)*	77 *(66.4%)*
Non-Smokers, *N*(%)	12 *(44.4%)*	23 *(25.8%)*	35 *(30.2%)*
Other, *N*(%)	0 *(0.0%)*	4 *(4.5%)*	4 *(3.4%)*
HbA1c (%), mean ± SD (*min-max*)	5.80 ± 0.60 *(4.6–8.0)*	5.44 ± 0.43 *(4.1–7.1)*	5.53 ± 0.50 *(4.1–8.0)*
Triglycerides (mg/dL), mean ± SD (*min-max*)	114.70 ± 65.08 *(39–278)*	92.39 ± 48.60 *(28–332)*	97.63 ± 53.47 *(28–332)*
Cholesterol (mg/dL), mean ± SD (*min-max*)	173.74 ± 43.95 *(120–282)*	179.51 ± 36.20 *(100–265)*	178.16 ± 38.03 *(100–282)*
HDL (mg/dL), mean ± SD (*min-max*)	68.44 ± 35.92 *(27–205)*	62.08 ± 18.85 *(29–118)*	63.57 ± 23.93 *(27–205)*
LDL (mg/dL), mean ± SD (*min-max*)	91.33 ± 40.89 *(30–217)*	99.58 ± 30.81 *(39–196)*	97.64 ± 33.44 *(30–217)*

*MMSE* Mini Mental State Examination, *HbA1c* Hemoglobin A1c, *HDL* high-density lipoprotein, *LDL* low-density lipoprotein, *SD* standard deviation, *min* minimum, *max* maximum

### Associations between hypertension and brainstem microstructure

**Fig. 1:**
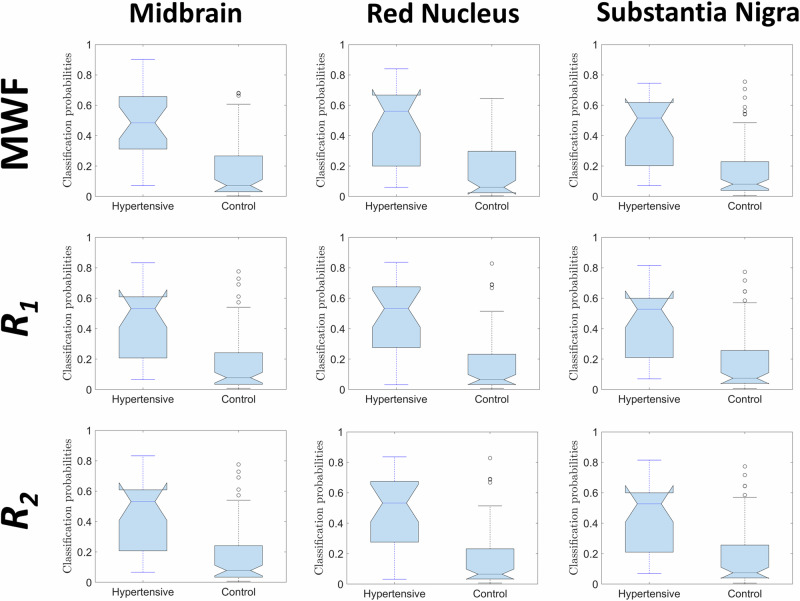
Box plots were employed to visually represent the probabilities calculated using the logistic regression method for both hypertensive and healthy control subjects across the midbrain, red nucleus and substantia nigra with respect to each MRI metric, including myelin water fraction (MWF), longitudinal relaxation rate (R_1_), and transverse relaxation rate (R_2_). A visual examination of the plots reveals a consistent pattern of notably lower probability of being hypertensive within the healthy control group compared to the hypertensive group

**Fig. 2:**
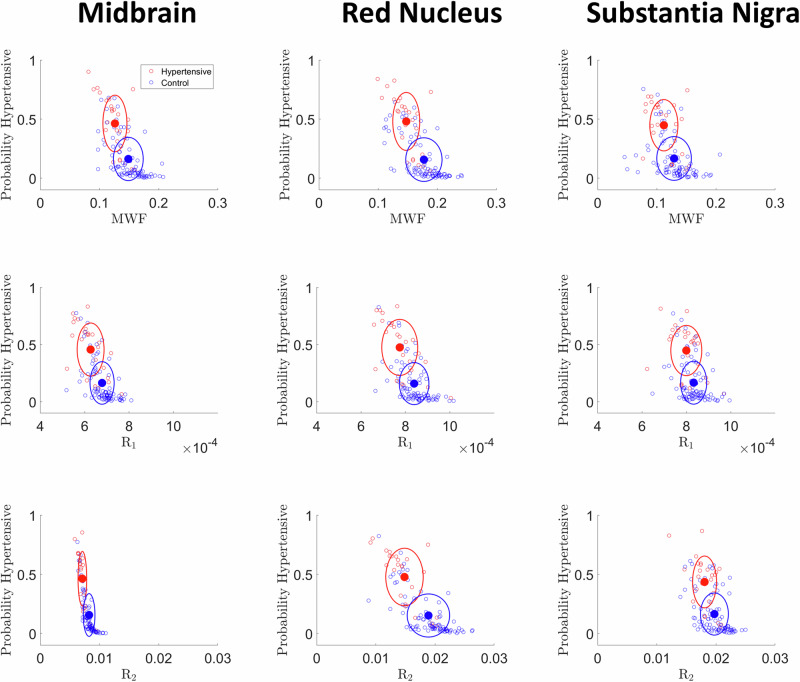
Plots illustrating the derived probabilities of hypertensive status based on the logistic regression model for MWF, R_1_ and R_2_. These results are presented for three distinct brainstem subregions: the midbrain, red nucleus, and substantia nigra. Within each plot, red is used to represent hypertensive subjects, while blue corresponds to healthy control subjects. Each plot features centroids represented by solid red and blue dots, which signify both the mean probability of hypertensive classification and the mean value of the respective MRI metric. Surrounding each centroid is a circle that indicates the corresponding standard deviation of the probability classification on the y-axis and the standard deviation of the respective MRI metric on the x-axis. MWF myelin water fraction, R_1_ longitudinal relaxation rate, R_2_ transverse relaxation rate

Comparing healthy control and hypertensive groups, Fig. 1 displays box plots of the probability values of hypertensive status across three representative ROIs, namely, the midbrain, red nucleus and substantia nigra, as determined by the logistic regression model. Results derived from all ROIs are shown in Supplementary Material. Visual examination of the plots consistently reveals that the prevalence of lower probability values associated with hypertensive status within the control group, as expected. These qualitative findings provide additional validation of the model’s robustness in capturing the distinctive characteristics between the hypertensive and control groups, specifically in terms of MWF, *R*_*1*_ and *R*_*2*_ values. Upon closer examination of individual ROIs, it becomes evident that some ROIs contain more discriminative information than others. Detailed findings in this regard are elaborated upon in Fig. 2 which presents plots illustrating the relationships between MWF, *R*_*1*_ and *R*_*2*_ and their corresponding probabilities of hypertensive status across the midbrain, red nucleus, and substantia nigra. These regions were chosen based on their superior discriminative abilities in distinguishing between healthy controls and hypertensive subjects, as determined through regression analysis. Results derived from all ROIs are shown in Supplementary Material. Each plot displays centroids positioned using the mean probability of hypertensive classification and the average value of the respective MRI metric. The standard deviation of the metric is incorporated as a radius in both x and y directions, resulting in distinct clusters that highlight differences between hypertensive subjects and controls. Visual inspection reveals that hypertensive subjects generally exhibit lower regional MWF, *R*_*1*_ and *R*_*2*_ values as compared to controls. This clustering is evident in both the x and y directions, signifying a clear differentiation in MWF, *R*_*1*_ and *R*_*2*_ values between the hypertensive and control groups, which further reinforces the model’s robustness.

**Fig. 3:**
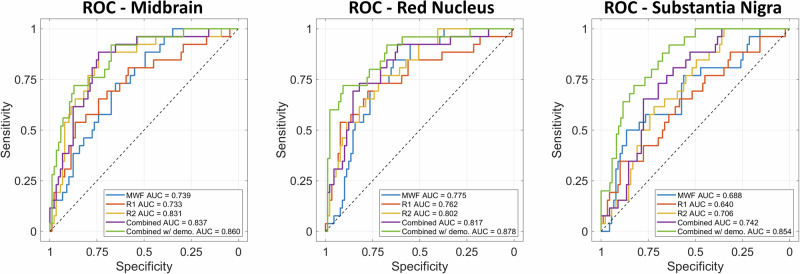
Receiver operating characteristic (ROC) curves for univariate classifiers and the multivariable classifier for relaxometry metrics (including all relaxometry measures, MWF, R_1_ and R_2_ as features) then relaxometry metrics when combined with demographics (including age, sex and HbA1c) for classifying the hypertension group. ROC curves are shown for three representative areas of the Midbrain, Red Nucleus and Substantia Nigra. Area under the curve (AUC) is displayed for each classifying group. MWF myelin water fraction, R_1_ longitudinal relaxation rate, R_2_ transverse relaxation rate. HbA1c Hemoglobin A1c

To further demonstrate the classification of hypertension based on our MRI metrics, Fig. 3 presents ROC curves for three representative brainstem substructures, namely, midbrain, red nucleus, and substantia nigra. The AUC values provide a quantitative measure of model performance, indicating good to very good performance for the univariate analysis of the relaxometry metrics alone (AUC = 0.640–0.831). Specifically, the lowest AUC was observed for *R*_*1*_ in the substantia nigra, while the highest AUC was observed for *R*_*2*_ in the midbrain. Classification performance improved when the relaxometry metrics were combined (AUC = 0.742–0.837) and were further improved when combined with demographic information of age, sex, and HbA1c (AUC = 0.854–0.878). The highest AUC values were achieved with the multivariable model, including demographic variables, in the red nucleus. These qualitative and quantitative observations underscore a strong association between hypertension and brainstem microstructural deterioration, including demyelination, within specific regions of interest. Overall, these findings suggest that our MRI metrics are effective (particularly when combined) in classifying hypertensive and healthy control subjects, thereby highlighting their potential utility in clinical settings for hypertension diagnosis.

Table 2 provides a summary of the results of the logistic regression analysis, examining the relationship between the hypertensive status and MWF, *R*_*1*_, *R*_*2*_, and age across 10 brainstem regions of interest. These findings are consistent with the trends illustrated in Figs. 1 and 2. Notably, significant or close-to-significant negative correlations were observed between the hypertensive status and MWF values in the medulla, midbrain, red nucleus, substantia nigra, subthalamic nucleus, and the middle cerebellar peduncles. Significant or close-to-significant negative correlations were also observed between hypertensive status and *R*_*1*_ in the medulla, midbrain, red nucleus and the substantia nigra. Significant or close-to-significant negative correlations between hypertensive status and *R*_*2*_ were found within the midbrain, red nucleus, and substantia nigra. HbA1c exhibited close-to-significant positive correlations in the medulla, midbrain and red nucleus when used with MWF, as well as close-to-significant positive correlations when used with *R*_*2*_. These correlations did not remain statistically significant following false discovery rate (FDR) correction apart from MWF and *R*_*2*_ in the red nucleus. While age, as anticipated, was a significant covariate of hypertensive status, showing significant positive slopes in relation to all metrics.

**Table 2:** Regression coefficients (standard error) and significance (*p*-value) (before, *p*, and after FDR correction, pFDR) of the hypertension status, HbA1c or age vs. relaxometry metrics (MWF, R_1_ and R_2_) derived from the logistic regression analysis

	MWF	HbA1c	Age	R1	HbA1c	Age	R2	HbA1c	Age
SCP	−0.34*(0.32)*, *p* = 0.289 *p*FDR = 0.311	0.42*(0.28)*, *p* = 0.133 *p*FDR = 0.189	1.16*(0.34)*, ***p*** < **0.001** ***p*FDR** < **0.001**	−0.40*(0.29)*, *p* = 0.162 *p*FDR = 0.226	0.36*(0.29)*, *p* = 0.210 *p*FDR = 0.274	1.20*(0.33)*, ***p*** < **0.001** ***p*FDR** < **0.001**	−0.43*(0.32)*, *p* = 0.184 *p*FDR = 0.448	0.33*(0.29)*, *p* = 0.256 *p*FDR = 0.256	1.17*(0.33)* ***p*** < **0.001** ***p*FDR** = **0.001**
MCP	−0.61*(0.32)*, ***p*** = **0.057** *p*FDR = 0.160	0.38*(0.28)*, *p* = 0.176 *p*FDR = 0.189	1.25*(0.33)*, ***p*** < **0.001** ***p*FDR** < **0.001**	−0.46*(0.29)*, *p* = 0.112 *p*FDR = 0.223	0.34*(0.28)*, *p* = 0.224 *p*FDR = 0.274	1.26*(0.33)*, ***p*** < **0.001** ***p*FDR** < **0.001**	−0.36*(0.30)*, *p* = 0.226 *p*FDR = 0.452	0.43*(0.28)*, *p* = 0.131 *p*FDR = 0.213	1.18*(0.33)*, ***p*** < **0.001** ***p*FDR** = **0.001**
ICP	−0.32*(0.29)*, *p* = 0.264 *p*FDR = 0.308	0.38*(0.28)*, *p* = 0.170 *p*FDR = 0.189	1.18*(0.34)*, ***p*** < **0.001** ***p*FDR** < **0.001**	−0.46*(0.29)*, *p* = 0.112 *p*FDR = 0.223	0.34*(0.29)*, *p* = 0.235 *p*FDR = 0.274	1.20*(0.33)*, ***p*** < **0.001** ***p*FDR** < **0.001**	−0.10*(0.28)*, *p* = 0.710 *p*FDR = 0.828	0.36*(0.28)*, *p* = 0.198 *p*FDR = 0.213	1.20*(0.34)*, ***p*** < **0.001** ***p*FDR** = **0.001**
CP	−0.33*(0.28)*, *p* = 0.232 *p*FDR = 0.296	0.39*(0.28)*, *p* = 0.163 *p*FDR = 0.189	1.20*(0.33)*, ***p*** < **0.001** ***p*FDR** < **0.001**	−0.41*(0.28)*, *p* = 0.136 *p*FDR = 0.223	0.34*(0.29)*, *p* = 0.233 *p*FDR = 0.274	1.26*(0.33)*, ***p*** < **0.001** ***p*FDR** < **0.001**	−0.22*(0.29)*, *p* = 0.437 *p*FDR = 0.664	0.39*(0.28)*, *p* = 0.169 *p*FDR = 0.213	1.13*(0.35)*, ***p*** = **0.001** ***p*FDR** = **0.002**
MED	−0.77*(0.38)*, ***p*** = **0.040** *p*FDR = 0.141	0.50*(0.28)*, ***p*** = **0.076** *p*FDR = 0.189	1.19*(0.33)*, ***p*** < **0.001** ***p*FDR** < **0.001**	−0.59*(0.30)*, ***p*** = **0.052** *p*FDR = 0.183	0.31*(0.29)*, *p* = 0.281 *p*FDR = 0.281	1.11*(0.33)*, ***p*** < **0.001** ***p*FDR** < **0.001**	−0.52*(0.36)*, *p* = 0.154 *p*FDR = 0.448	0.41*(0.29)*, *p* = 0.161 *p*FDR = 0.213	0.97*(0.37)*, ***p*** = **0.008** ***p*FDR** = **0.010**
MID	−0.76*(0.34)*, ***p*** = **0.028** *p*FDR = 0.223	0.50*(0.29)*, ***p*** = **0.086** *p*FDR = 0.189	0.97*(0.34)*, ***p*** = **0.004** ***p*FDR** = **0.005**	−0.64*(0.31)*, ***p*** = **0.038** *p*FDR = 0.123	0.38*(0.29)*, *p* = 0.189 *p*FDR = 0.274	1.03*(0.33)*, ***p*** = **0.001** ***p*FDR** = **0.001**	-1.24*(0.52)*, ***p*** = **0.018** *p*FDR = 0.123	0.45*(0.30)*, *p* = 0.136 *p*FDR = 0.213	0.47*(0.41)*, *p* = 0.259 *p*FDR = 0.259
PON	−0.46*(0.31)*, *p* = 0.143 *p*FDR = 0.223	0.41*(0.27)*, *p* = 0.131 *p*FDR = 0.189	1.16*(0.33)*, ***p*** < **0.001** ***p*FDR** < **0.001**	−0.43*(0.29)*, *p* = 0.143 *p*FDR = 0.223	0.32*(0.28)*, *p* = 0.261 *p*FDR = 0.281	1.21*(0.33)*, ***p*** < **0.001** ***p*FDR** < **0.001**	−0.25*(0.34)*, *p* = 0.474 *p*FDR = 0.664	0.40*(0.28)*, *p* = 0.150 *p*FDR = 0.213	1.12*(0.36)*, ***p*** = **0.002** ***p*FDR** = **0.003**
RN	−0.89*(0.33)*, ***p*** = **0.007** ***p*FDR** = **0.092**	0.53*(0.29)*, ***p*** = **0.068** *p*FDR = 0.189	0.95*(0.34)*, ***p*** = **0.005** ***p*FDR** = **0.005**	−0.76*(0.31)*, ***p*** = **0.015** *p*FDR = 0.155	0.38*(0.30)*, *p* = 0.206 *p*FDR = 0.274	1.08*(0.33)*, ***p*** = **0.001** ***p*FDR** < **0.001**	−0.92*(0.34)*, ***p*** = **0.006** ***p*FDR** = **0.090**	0.50*(0.29)*, ***p*** = **0.085** *p*FDR = 0.213	0.77*(0.34)*, ***p*** = **0.025** ***p*FDR** = **0.027**
SN	−0.52*(0.31)*, ***p*** = **0.096** *p*FDR = 0.189	0.47*(0.29)*, *p* = 0.103 *p*FDR = 0.189	1.11*(0.33)*, ***p*** < **0.001** ***p*FDR** = **0.001**	−0.53*(0.29)*, ***p*** = **0.066** *p*FDR = 0.185	0.37*(0.28)*, *p* = 0.196 *p*FDR = 0.274	1.24*(0.33)*, ***p*** < **0.001** ***p*FDR** < **0.001**	−0.56*(0.31)*, *p* = 0.070 *p*FDR = 0.328	0.47*(0.30)*, *p* = 0.114 *p*FDR = 0.213	1.10*(0.34)*, ***p*** = **0.001** ***p*FDR** = **0.002**
STH	−0.65*(0.31)*, ***p*** = **0.034** *p*FDR = 0.141	0.45*(0.31)*, *p* = 0.144 *p*FDR = 0.189	1.10*(0.33)*, ***p*** < **0.001** ***p*FDR** = **0.001**	−0.20*(0.27)*, *p* = 0.452 *p*FDR = 0.452	0.37*(0.28)*, *p* = 0.185 *p*FDR = 0.274	1.25*(0.33)*, ***p*** < **0.001** ***p*FDR** < **0.001**	0.04*(0.25)*, *p* = 0.858 *p*FDR = 0.858	0.37*(0.28)*, *p* = 0.185 *p*FDR = 0.213	1.25*(0.33)*, ***p*** < **0.001** ***p*FDR** = **0.001**

Results are shown for the 10 brainstem subregions investigated. Bolded p-values indicate statistical significance (*p* < 0.05) or close-to-significance (*p* < 0.1)

*HbA1c* hemoglobin A1c, *MWF* myelin water fraction, *R*_*1*_ Longitudinal relaxation rate, *R*_*2*_ Transverse relaxation rate, *ROIs* regions-of-interest, *SCP* superior cerebellar peduncle, *MCP* middle cerebellar peduncle, *ICP* inferior cerebellar peduncle, *CP* cerebral peduncle, *MED* medulla, *MID* midbrain, *PON* pons, *RN* red nucleus, *SN* substantia nigra, *STH* subthalamic nucleus

## Discussion

In this qMRI study utilizing multicomponent relaxometry, we showed a significant association between deteriorated brainstem microstructure, indicated by lower MWF, R_1_, or R_2_ values, and hypertensive status across multiple brainstem subregions in a cohort of 116 cognitively unimpaired adults. Notably, our findings establish a novel direct link between brainstem myelin content, measured by MWF, and hypertensive status. This correlation resonates with previous research, reinforcing the idea that brainstem microstructural disruptions are likely tied to cardiovascular risk factors, particularly hypertension. Our results provide new insights into the complex relationship between brainstem integrity and blood pressure regulation, underscoring the potential for MRI-based biomarkers to aid in the early detection and monitoring of hypertension-related neural changes.

The brainstem plays a crucial role in regulating blood pressure through its control of autonomic functions [59]. Hypertension is closely linked to reduced cerebral blood flow, which can impair brain function by limiting the supply of oxygen, glucose, and nutrients [60]. This reduction in blood flow has a cascading effect, impacting the function of oligodendrocytes, the glial cells responsible for maintaining myelin integrity [61]. When oligodendrocytes struggle to maintain myelin due to diminished blood flow, neural signal transmission in the brainstem is compromised [62]. Myelin is particularly essential in the brainstem, facilitating rapid and precise neural communication. This creates a feedback loop where brainstem damage leads to disrupted blood pressure regulation, perpetuating a cycle of physiological disturbances. Further longitudinal studies are necessary to confirm the connection between myelin content and hypertension, but this research suggests a critical relationship between brainstem integrity and blood pressure control.

Our study revealed a significant association between MWF, R_1_, and R_2_ values and hypertensive status in the midbrain, particularly in the midbrain tegmentum, including the substantia nigra and red nucleus. The midbrain tegmentum is known for its role in motor control, pain modulation, and sensory processing, but also contains key components of the brain’s dopaminergic system, which influences autonomic functions, including blood pressure regulation [63–66]. The ventral tegmental area, a region within the midbrain tegmentum, sends dopaminergic and GABAergic projections to brainstem regions crucial for cardiovascular regulation, such as the nucleus tractus solitarius and rostral ventrolateral medulla [67–69]. The substantia nigra itself has been implicated in blood pressure control [70]. We propose that the midbrain tegmentum modulates sympathetic and parasympathetic activity to influence blood pressure homeostasis, and disruptions in this balance may contribute to hypertension. Notably, while the medulla showed a significant association between MWF and hypertensive status, it did not show a significant association with R_2_, possibly due to the protective effects of the paramedian and lateral bulbar arteries, which supply the medulla and may shield it from ischemic damage [71].

Currently, peripheral blood pressure is the sole biomarker used in clinical practice for diagnosing and managing hypertension [72]. While blood pressure measurement is valuable, it is a sensitive but non-specific indicator of cardiovascular function. Consequently, many patients undergoing antihypertensive treatment fail to achieve their blood pressure targets [73]. By leveraging modern diagnostic techniques to categorize patients’ physiological phenotypes, such as pulse wave velocity, renal function, and advanced imaging methods like MWF, clinicians can gain enhanced specificity. This can enable personalized treatment approaches tailored to individual patients’ needs, potentially leading to more effective management of hypertension.

While our study involved a substantial cohort and utilized advanced MRI techniques to probe brainstem microstructural integrity and regional myelin content, it is important to acknowledge inherent limitations in our study. The cross-sectional nature of our design prevents the establishment of causal relationships between demyelination and hypertensive status. To validate this potential link, future prospective longitudinal studies are essential. Moreover, unraveling the causal connection between hypertension and myelination is challenging due to the often-present array of cardiovascular risk factors. Elucidating the link between how antihypertensive medication may have a protective effect on the microstructure of the brainstem is additionally an avenue that requires further study. Given the cohort’s size, it’s unfeasible to encompass all these factors in our logistic model. Further, the potential impact of anti-hypertensive medication duration on our results was not investigated as we do not have access to information on the length of time participants have been taking these medications. Future studies should consider collecting and analyzing this important variable to provide a more comprehensive understanding of the relationship between hypertension treatment and myelin integrity. Finally, the determination of MR parameters can be influenced by various biological and methodological elements, including magnetization transfer, water pool exchange, J-coupling, off-resonance effects, spin locking, water diffusion, flow, temperature, hydration, internal gradients, and structural complexities like fiber arrangements or intersections [39].

## Supplementary information

Supporting Information
