# Supplementary material for: Investigating the association between human brainstem microstructural integrity and hypertension using magnetic resonance relaxometry
Source: Hypertens Res. 2025 Jan 23;48(4):1564–74. doi: 10.1038/s41440-025-02114-1 (PMC11972960; doi:10.1038/s41440-025-02114-1)
Supplement: Supplementary file 1 — Supporting Information [file 41440_2025_2114_MOESM1_ESM.pdf]

Supporting Information for

Investigating the association between human brainstem microstructural degeneration and hypertension using multicomponent magnetic resonance relaxometry.

John P. Laporte, Mohammad A.B.S. Akhonda, Luis E. Cortina, Mary E. Faulkner, Zhaoyuan Gong, Alex Guo, Jonghyun Bae, Noam Fox, Nathan Zhang, Christopher M. Bergeron, Luigi Ferrucci, Josephine M. Egan, and Mustapha Bouhrara

Corresponding author: Mustapha Bouhrara
Email: bouhraram@mail.nih.gov

This PDF file includes:

Figures S1 to S7

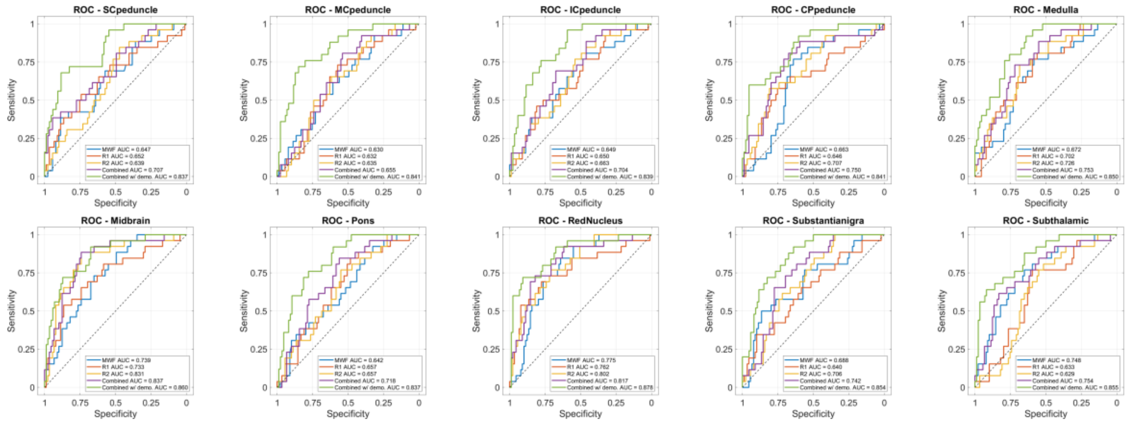

Fig. S1. Receiver operating characteristic (ROC) curves for univariate classifiers and the multivariable classifier for relaxometry metrics (including all relaxometry measures, MWF, R_1 and R_2 as features) then relaxometry metrics when combined with demographics (including age, sex and HbA1c) for classifying the hypertension group. ROC curves are shown for the ten ROIs investigated. Area under the curve (AUC) is displayed for each classifying group.

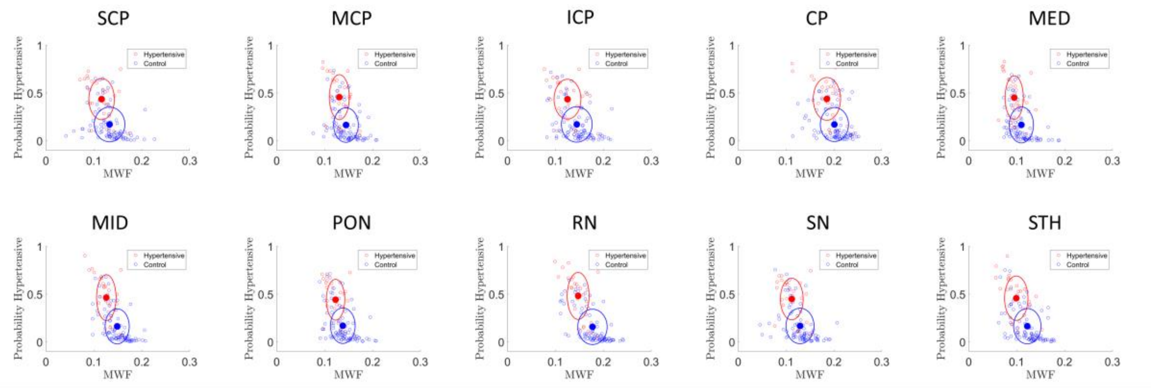

Fig. S2. Plots illustrating the derived probabilities of hypertensive status based on the logistic regression model for MWF. These results are presented for ten distinct brainstem ROIs. Within each plot, red is used to represent hypertensive subjects, while blue corresponds to healthy control subjects. Each plot features centroids represented by solid red and blue dots, which signify both the mean probability of hypertensive classification and the mean value of the respective MRI metric. Surrounding each centroid is a circle that indicates the corresponding standard deviation of the probability classification on the y-axis and the standard deviation of the respective MRI metric on the x-axis.

Notes. ROI, region-of-interest; MWF, myelin water fraction; SCP, superior cerebellar peduncle; MCP, middle cerebellar peduncle; ICP, inferior cerebellar peduncle; CP, cerebral peduncle; MED, medulla; MID, midbrain; PON, pons; RN, red nucleus; SN, substantia nigra; STH, subthalamic nucleus

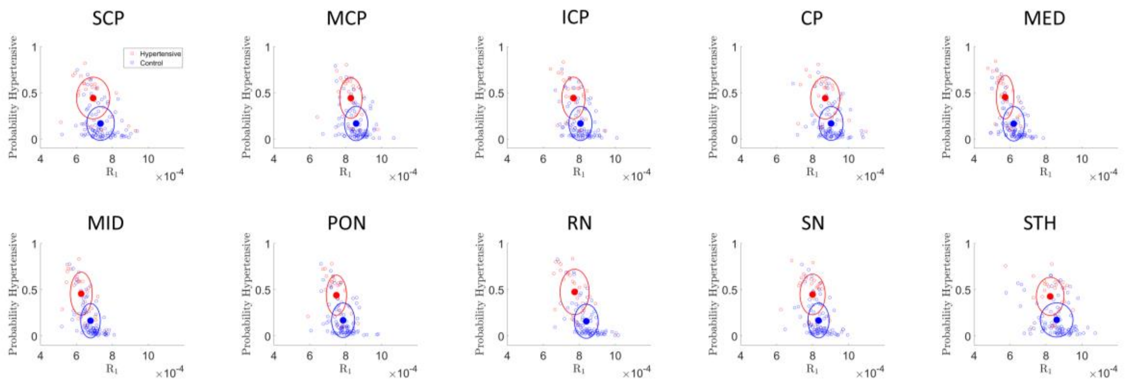

Fig. S3. Plots illustrating the derived probabilities of hypertensive status based on the logistic regression model for R_1. These results are presented for ten distinct brainstem ROIs. Within each plot, red is used to represent hypertensive subjects, while blue corresponds to healthy control subjects. Each plot features centroids represented by solid red and blue dots, which signify both the mean probability of hypertensive classification and the mean value of the respective MRI metric. Surrounding each centroid is a circle that indicates the corresponding standard deviation of the probability classification on the y-axis and the standard deviation of the respective MRI metric on the x-axis.

Notes. ROI, region-of-interest; R_1, longitudinal relaxation rate; SCP, superior cerebellar peduncle; MCP, middle cerebellar peduncle; ICP, inferior cerebellar peduncle; CP, cerebral peduncle; MED, medulla; MID, midbrain; PON, pons; RN, red nucleus; SN, substantia nigra; STH, subthalamic nucleus

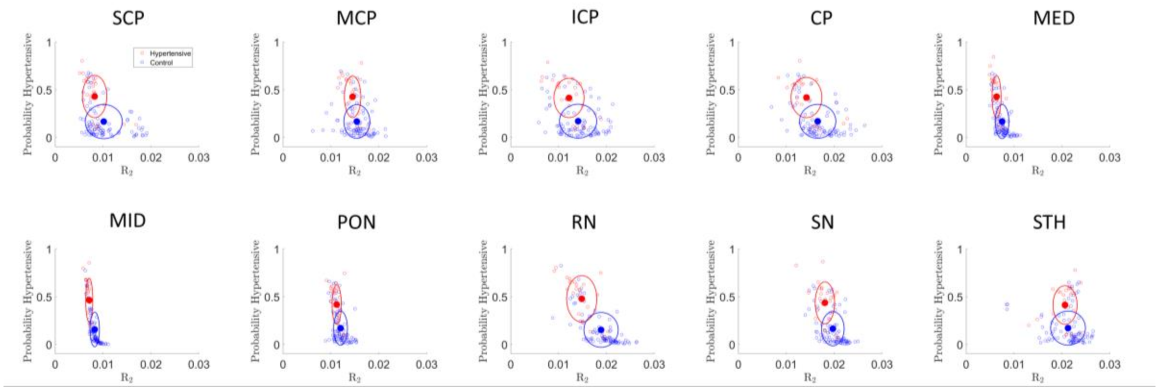

Fig. S4. Plots illustrating the derived probabilities of hypertensive status based on the logistic regression model for R_2. These results are presented for ten distinct brainstem ROIs. Within each plot, red is used to represent hypertensive subjects, while blue corresponds to healthy control subjects. Each plot features centroids represented by solid red and blue dots, which signify both the mean probability of hypertensive classification and the mean value of the respective MRI metric. Surrounding each centroid is a circle that indicates the corresponding standard deviation of the probability classification on the y-axis and the standard deviation of the respective MRI metric on the x-axis.

Notes. ROI, region-of-interest; R_2, transverse relaxation rate; SCP, superior cerebellar peduncle; MCP, middle cerebellar peduncle; ICP, inferior cerebellar peduncle; CP, cerebral peduncle; MED, medulla; MID, midbrain; PON, pons; RN, red nucleus; SN, substantia nigra; STH, subthalamic nucleus

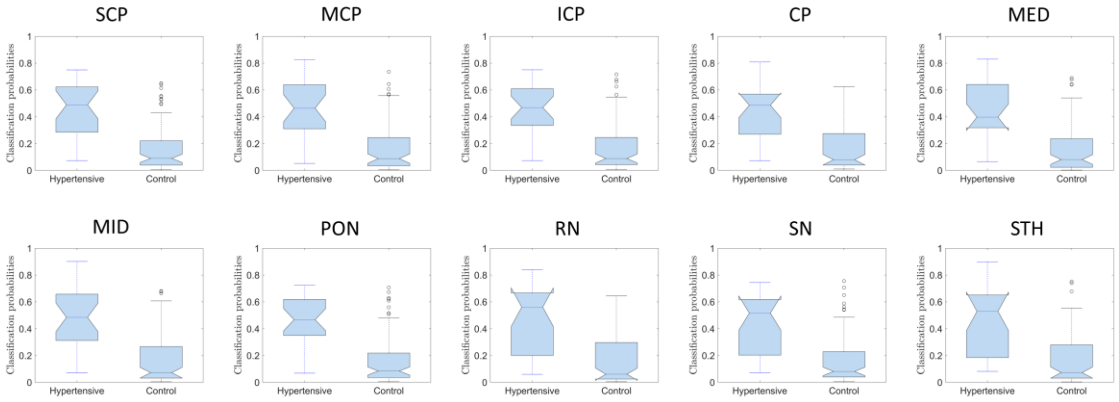

Fig. S5. Box plots visualizing probability values as determined by the logistic regression model for both hypertensive and healthy control subjects across the ten brainstem ROIs with respect to MWF. A visual examination of the plots reveals a consistent pattern of notably lower probability of being hypertensive within the healthy control group compared to the hypertensive group.

Notes. ROI, region-of-interest; MWF, myelin water fraction; SCP, superior cerebellar peduncle; MCP, middle cerebellar peduncle; ICP, inferior cerebellar peduncle; CP, cerebral peduncle; MED, medulla; MID, midbrain; PON, pons; RN, red nucleus; SN, substantia nigra; STH, subthalamic nucleus

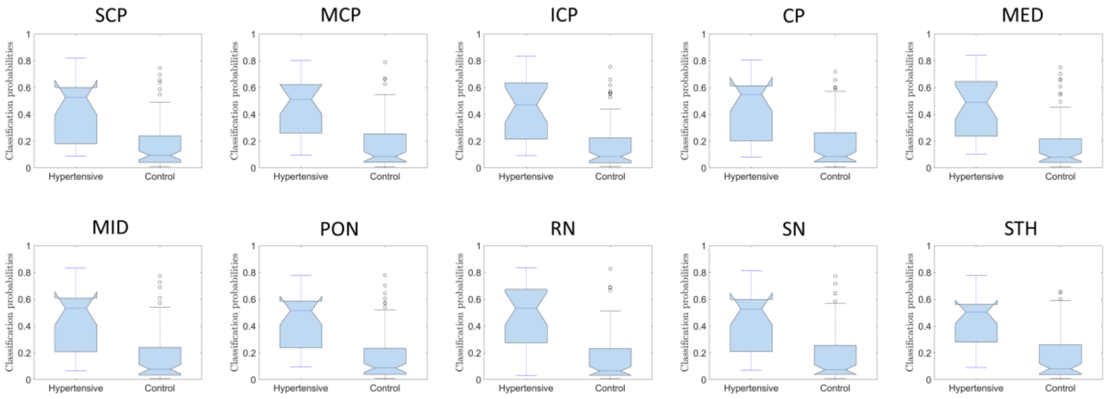

Fig. S6. Box plots visualizing probability values as determined by the logistic regression model for both hypertensive and healthy control subjects across the ten brainstem ROIs with respect to R_1. A visual examination of the plots reveals a consistent pattern of notably lower probability of being hypertensive within the healthy control group compared to the hypertensive group.

Notes. ROI, region-of-interest; R_1, longitudinal relaxation rate; SCP, superior cerebellar peduncle; MCP, middle cerebellar peduncle; ICP, inferior cerebellar peduncle; CP, cerebral peduncle; MED, medulla; MID, midbrain; PON, pons; RN, red nucleus; SN, substantia nigra; STH, subthalamic nucleus

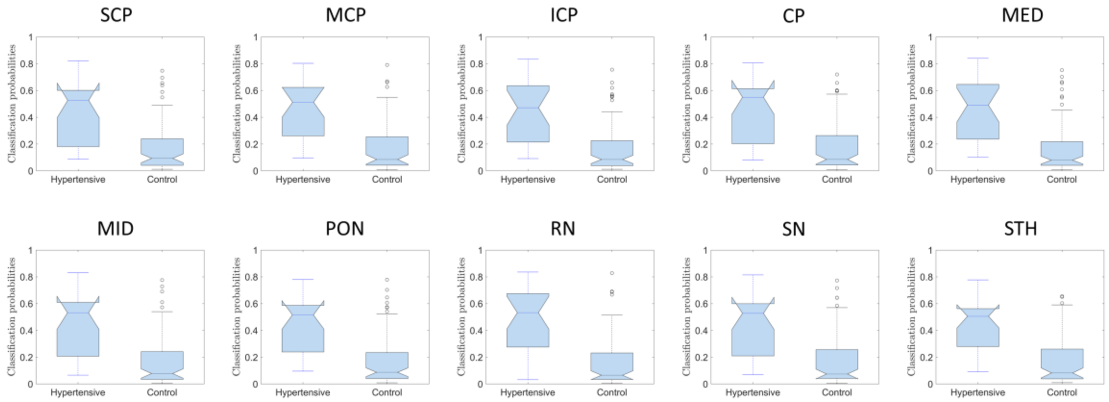

Fig. S7. Box plots visualizing probability values as determined by the logistic regression model for both hypertensive and healthy control subjects across the ten brainstem ROIs with respect to R_2. A visual examination of the plots reveals a consistent pattern of notably lower probability of being hypertensive within the healthy control group compared to the hypertensive group.

Notes. ROI, region-of-interest; R_2, transverse relaxation rate; SCP, superior cerebellar peduncle; MCP, middle cerebellar peduncle; ICP, inferior cerebellar peduncle; CP, cerebral peduncle; MED, medulla; MID, midbrain; PON, pons; RN, red nucleus; SN, substantia nigra; STH, subthalamic nucleus
